# Development of a Comprehensive Hospital-Based Elder Abuse Intervention: An Initial Systematic Scoping Review

**DOI:** 10.1371/journal.pone.0125105

**Published:** 2015-05-04

**Authors:** Janice Du Mont, Sheila Macdonald, Daisy Kosa, Shannon Elliot, Charmaine Spencer, Mark Yaffe

**Affiliations:** 1 Women’s College Research Institute, Women’s College Hospital, Toronto, Ontario, Canada; 2 Dalla Lana School of Public Health Sciences, University of Toronto, Toronto, Ontario, Canada; 3 Ontario Network of Sexual Assault/Domestic Violence Treatments Centres, Toronto, Ontario, Canada; 4 Gerontology Research Centre, Simon Fraser University, Burnaby, British Columbia, Canada; 5 Department of Family Medicine, McGill University, Montreal, Québec, Canada; 6 Department of Family Medicine, St. Mary’s Hospital Centre, Montreal, Québec, Canada; Cardiff University, UNITED KINGDOM

## Abstract

**Introduction:**

Elder abuse, a universal human rights problem, is associated with many negative consequences. In most jurisdictions, however, there are no comprehensive hospital-based interventions for elder abuse that address the totality of needs of abused older adults: psychological, physical, legal, and social. As the first step towards the development of such an intervention, we undertook a systematic scoping review.

**Objectives:**

Our primary objective was to systematically extract and synthesize actionable and applicable recommendations for components of a multidisciplinary intersectoral hospital-based elder abuse intervention. A secondary objective was to summarize the characteristics of the responses reviewed, including methods of development and validation.

**Methods:**

The grey and scholarly literatures were systematically searched, with two independent reviewers conducting the title, abstract and full text screening. Documents were considered eligible for inclusion if they: 1) addressed a response (e.g., an intervention) to elder abuse, 2) contained recommendations for responding to abused older adults with potential relevance to a multidisciplinary and intersectoral hospital-based elder abuse intervention; and 3) were available in English.

**Analysis:**

The extracted recommendations for care were collated, coded, categorized into themes, and further reviewed for relevancy to a comprehensive hospital-based response. Characteristics of the responses were summarized using descriptive statistics.

**Results:**

649 recommendations were extracted from 68 distinct elder abuse responses, 149 of which were deemed relevant and were categorized into 5 themes: Initial contact; Capacity and consent; Interview with older adult, caregiver, collateral contacts, and/or suspected abuser; Assessment: physical/forensic, mental, psychosocial, and environmental/functional; and care plan. Only 6 responses had been evaluated, suggesting a significant gap between development and implementation of recommendations.

**Discussion:**

To address the lack of evidence to support the recommendations extracted in this review, in a future study, a group of experts will formally evaluate each recommendation for its inclusion in a comprehensive hospital-based response.

## Introduction

Elder abuse, a universal human rights problem [1], is often defined as the mistreatment of older adults through “actions/behaviours or lack of actions/behaviours that cause harm or risk of harm within a trust relationship” [2](p.2). According to the United States Department of Justice [3], examples of abuse of older adults can include isolation and neglect by an adult child or caregiver; physical or sexual assault by an intimate partner, adult child or caregiver; financial or material exploitation by a stranger, family member or professional; abuse or neglect by a partner with advancing dementia; and/or systemic neglect by a long-term care provider resulting in inadequate services. Although many forms of abuse appear unlawful and involvement of criminal justice systems may be appropriate, perpetrators are rarely prosecuted and future offenses are thereby not deterred [4,5].

A growing research literature on elder abuse suggests that the problem is widespread. Cooper, Selwood, and Livingston [6] systematically reviewed studies measuring its prevalence and found globally that in general populations rates ranged between 3.2% to 27.5%. When assessing for specific types of abuse 4.2% of older adults reported psychological abuse, 0.5% to 4.3% physical abuse, 1.1 to 10.8% verbal abuse, 1.3 to 5.0% financial abuse, and 0.2 to 6.7 neglect. Older adults who are cognitively impaired, socially isolated, and very elderly (e.g., over age 75 or 80) or who have a lower educational status and a lower income are at an increased risk (for different types) of elder abuse [7–9]. The problem of elder abuse will continue to grow in magnitude as the population ages; globally, the number of people aged 80 years and older will almost quadruple to 395 million between 2000 and 2050 [10].

Elder abuse is associated with many negative health outcomes. Studies have shown that it is a notable source of emotional distress, depression, anxiety, social isolation, as well as loss of financial resources for self-care [11] and can result in immediate physical injuries, sexually transmitted infections, chronic health problems, and death directly and indirectly related to the abuse [12,13]. Moreover, abused older adults are more likely than those not abused to report higher levels of lung, bone, joint and digestive problems, chronic pain, and psychological issues such as depression, anxiety, and post-traumatic stress disorder [14,15]. Among community dwelling older adults, elder abuse is also associated with increased rates of emergency department use [16], admission to nursing facilities [14], and hospitalization [17,18].

The prevalence and adverse outcomes of elder abuse call for further clarity surrounding the role that health professionals might play in responding to the issue. Although elder abuse is increasingly seen as being within the scope of medical practice, a review of the scientific literature revealed that the time and resources needed to address such a complex issue are increasingly constrained across all health systems [19]. Few elder abuse interventions are housed in hospitals and physicians frequently do not assess for or identify elder abuse because for the most part it has not been a component of their training [20]. Internationally, there is growing recognition that to adequately and appropriately address such a multifaceted issue, health providers will need to work collaboratively with the social welfare sector (e.g., to provide housing, financial, and legal supports) [21]. The problem lies in that in most jurisdictions there is currently no comprehensive hospital-based intervention for elder abuse that addresses the totality of needs of abused older adults: psychological, physical, legal, and social.

Forensic nurse examiner hospital-based violence programs, often in collaboration with community agencies and law enforcement services, have played a key role in providing comprehensive health, psychosocial, and medico-legal care to victims of sexual assault that present in the emergency department so as to minimize harm experienced and reduce the likelihood of future victimizations [22]. Generally, mandates of forensic nurse-examiner hospital-based violence programs do not include elder abuse. Of 754 forensic nurse examiner programs in the United States listed with the International Association of Forensic Nurses, only 58 have reported having staff who can provide medical/legal forensic examination for elder abuse and neglect [23]. In Ontario, Canada, where there are 35 such programs, no comprehensive response to the various types of elder abuse currently exists, although over 80% of program leaders surveyed expressed interest in expanding their mandates to work collaboratively with other services in the community (e.g., Public Trustee and Guardian) to address this issue [24].

To fill the gap in service provision to abused older adults and build on the success, infrastructure, and expertise of forensic nurse examiner programs, we undertook a systematic scoping review of the scholarly and grey literatures as the first steps towards the development of a multidisciplinary and intersectoral hospital-based elder abuse intervention. This methodology was utilized to capture the breadth of the available recommendations [25,26] relevant to addressing the complexity of elder abuse within a comprehensive hospital-based response. Our primary objective was to systematically extract and synthesize actionable and applicable recommendations for components of a hospital-based elder abuse intervention. A secondary objective of this systematic scoping review was to summarize the characteristics of the responses reviewed, including their methods of development and validation.

## Methods

This review was conducted in accordance with PRISMA guidelines (see S1 Appendix).

### Data sources and search strategy

We employed a systematic search strategy and data extraction methodology to ensure scientific rigour. With the assistance of an experienced medical librarian, the scholarly literature was searched using the electronic databases Medline, Embase, and PsychInfo from January 1, 1995 to October 11, 2013. Search terms included elder abuse, elder neglect, elder mistreatment, elder maltreatment, intervention, response, guideline, protocol, consensus, and recommendation (see S2 Appendix. Hospital-based Elder Abuse Intervention Systematic Scoping Review Search Strategy). The grey literature search was concluded December 6, 2013 and included a targeted examination of a total of 252 guideline databases (e.g., National Guideline Clearinghouse) and websites focused on elder abuse (e.g., National Center on Elder Abuse), interpersonal violence (e.g., Women Against Violence Europe), and aging and care for older persons (add e.g., Aging in America). Where the website search function allowed for Boolean operators to combine or exclude keywords (e.g., AND, OR, NOT, or AND NOT), the search statement was run as: ("Elder abuse" OR "elder maltreatment" OR "elder mistreatment" OR "older persons abuse") AND (protocols OR guidelines OR practices OR "consensus statement") AND (intervention OR response). Where Boolean operators could not be accommodated, key words were run individually. A search of Google was run using the same search statement to find any relevant documents that may have been missed in the targeted search. The first 100 search results (approximately 10 pages) were reviewed for any relevance/inclusion. During full text review of all eligible documents, other potentially relevant documents cited were retrieved and reviewed where possible.

### Document inclusion/exclusion criteria

Documents were considered eligible for inclusion if they: 1) addressed a response to elder abuse; 2) contained recommendations for responding to abused older adults with potential relevance to a multidisciplinary and intersectoral hospital-based elder abuse intervention; and 3) were available in English. Documents were excluded if the focus was solely on elder self-neglect, were not free-of-cost, were web pages only, were curricula, and/or were screening tools.

### Document selection

**Fig 1:**
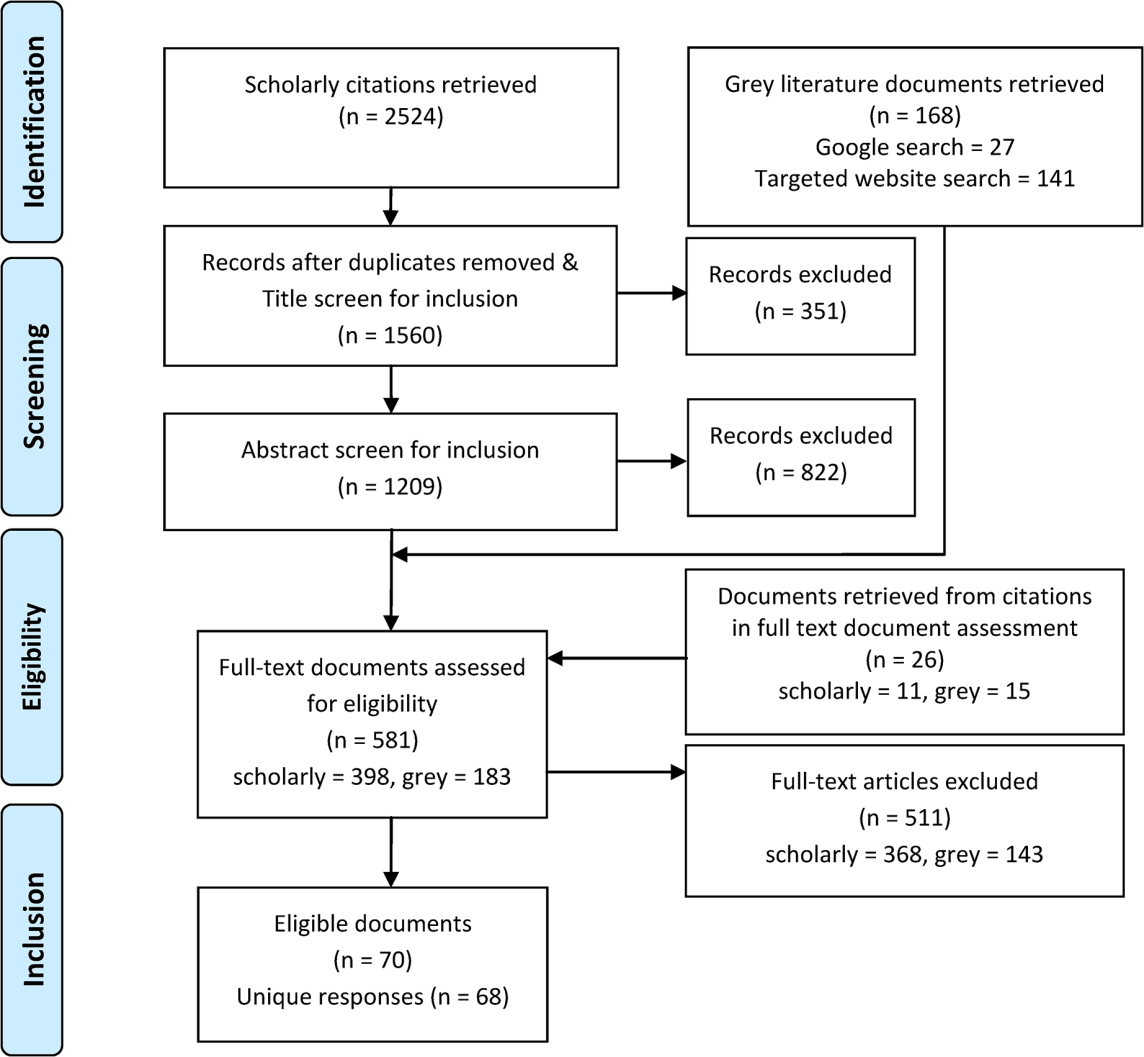
PRISMA Flow Diagram for the Identification of Elder Abuse Responses.

Two independent reviewers conducted the title, abstract, and full text screening (JDM, MW). Documents were retained at each stage of screening if the inclusion criteria were met (see **Fig 1**.). Disagreements were resolved through discussion and consensus.

### Data abstraction

A data extraction form was created by the research team to record the characteristics of the included documents/responses: name, year of publication, country of publication, intended sector, stakeholder involvement, method of development, and method of validation (see S1 Dataset). Recommendations, defined as strong declarative statements [27] that were actionable and applicable by a multidisciplinary intersectoral team of professionals in a comprehensive hospital-based elder abuse intervention, as determined by the research team, were collected in a separate excel table. Four authors (JDM, SM, DK, SE) independently piloted the data extraction form, modifications and clarifications to the form were made where necessary, to achieve consensus in data extraction, which was then performed in dependently by two reviewers (DK, SE). Data extraction disagreements were resolved by discussion and consensus, and a third author (JDM) was consulted where an agreement could not be reached. Kappa statistics were generated to evaluate consistency in extraction of the data. For various characteristics of the approaches examined, the kappa values ranged from 0.676 to 1.00 (moderate to perfect agreement).

### Data synthesis and analysis

Characteristics of the responses were summarized using descriptive statistics. The extracted recommendations for care were collated, coded, and categorized into themes over several consensus meetings (JDM, SM, SK, SE). Recommendations within themes were then further reviewed for relevancy to hospital-based forensic nurse examiner models of care (JDM, DK, SE), under the direction of the Provincial Coordinator of Ontario’s 35 Sexual Assault/Domestic Violence Treatment Centres (SM), who has over 20 years’ experience as a forensic nurse examiner providing care to victims of violence. Duplicate or similar recommendations and those that provided additional detail to a broader more general recommendation were removed. Only those recommendations pertaining to the ‘what’ should be included in the hospital-based response were reported in this systematic review (e.g., “Determine the level and urgency of safety concerns” [28], whereas those recommendations pertaining more to the ‘how’ to provide care (e.g., “When asking questions, talk to the older person alone, don’t rely on the explanation of others, use non-threatening words and questions”) [29] were retained for future use in the development of curricula and training tools.

## Results

Two thousand five hundred twenty-four scholarly citations were retrieved, along with 168 grey literature documents, 141 from the website and guideline database searches, and 27 from Google search. After removing duplicate citations, screening titles and abstracts of the scholarly literature, and adding additional documents based on citations seen during full text review, 581 full text documents were reviewed, 70 of which were eligible for inclusion in this review, based on our inclusion and exclusion criteria. During full text review, two documents each were combined where they represented aspects of the same response, for a final 68 distinct elder abuse responses reviewed. Documents that were part of a larger ‘parent’ document or drew heavily from a larger ‘parent’ document were excluded. Where a more recent version of a document by the same authors was available, the updated version was reviewed (**Fig 1**.).

### Characteristics of the included responses to elder abuse

Of the 68 responses reviewed, 28 were categorized as guidelines, 18 as frameworks, seven as protocols (including a subchapter of protocol), six as manuals (including subchapter of a manual), four as tools, three as interventions, and two as tool kits. Responses were categorized as self-identified where possible. Where the response did not self-identify, two authors (JDM, SK) categorized them based on their mission statement or other relevant content. Three of the included responses were primarily focused on the abuse of vulnerable adults, but also included abuse of the elderly [29–31]. Most of the responses were published in the United States (53%), followed by Canada (32%), Australia (6%), the United Kingdom (3%), Portugal (3%), New Zealand (1%), and Hong Kong (1%). Approximately half (49%) were targeted to more than one sector: 79% the health sector, 59% the community/social service sector, 31% the legal sector, 28% the law enforcement sector, 10% the financial sector, and 10% other sectors (e.g., faith-based institutions/spiritual leaders) (see Table 1).

**Table 1:** Characteristics of the Responses to Elder Abuse.

Response Name, Publication Date	Country*	Target Sector**				
		H	C/S	F	LE	L
A Community Resource Guide for Service Providers, 2012 [53]	CAN	✓	✓			
A Guide for Elder Abuse Protocols: Developed for Community Service Organisations, no date [54]	AUS		✓			
A Model Intervention for Elder Abuse and Dementia, 2000 [33]	USA		✓			
A Resource for Service Providers working with Older Women Experiencing Abuse, 2009 [55]	CAN	✓	✓			
Abuse and Neglect of an Older or Vulnerable Person, 2006 [56]	CAN	✓	✓		✓	✓
Abuse Prevention of Older Adults Network Peterborough: Coordinated Community Response Agreement, 2005 [29]	CAN	✓	✓	✓	✓	✓
Abuse/maltreatment of Older Adults: A Guideline for Counselors, 2000 [57]	USA		✓			
ACT Elder Abuse Prevention Program Policy, 2012 [58]	AUS	✓	✓		✓	
Act on Adult Abuse and Neglect: A Manual for Vancouver Coastal Health Staff, no date [30]	CAN	✓	✓			
Adult Protective Services Protocol, 2013 [59]	USA	✓	✓	✓		✓
Adult Protective Services: Financial Exploitation, 2010 [60]	USA		✓			
Adult Victims of Abuse Protocols, 2005 [61]	CAN	✓	✓		✓	✓
An Elder Abuse Resource and Intervention Guide, 1995 [62]	CAN	✓	✓		✓	✓
Burn Injuries Inflicted on Children or the Elderly: A Framework for Clinical and Forensic Assessment, 2005 [63]	USA	✓				
Calgary's Action Group on Elder Abuse: Elder Abuse Protocol, 2007 [64]	CAN	✓	✓		✓	✓
Can you Spot the Signs of Elder Mistreatment?, 1999 [65]	USA	✓				
Clinician's Role in the Documentation of Elder Mistreatment, 2009 [66]	USA	✓				
Decision Tree for the Detection and Treatment of Financial Exploitation of Older Adults, 2013 [67]	USA	✓				
Effectively Detect and Manage: Elder Abuse, 2004 [68]	USA	✓				
Elder Abuse and Women's Health, 2013 [69]	USA	✓				
Elder Abuse Assessment and Intervention—Reference Guide, 2010 [70]	CAN	✓	✓			
Elder Abuse Assessment Tool Kit, 2011 [71]	CAN	✓	✓		✓	
Elder Abuse Detection and Intervention: A Collaborative Approach, 2007 [72]	USA	✓	✓	✓	✓	✓
Elder Abuse Diagnosis and Intervention (EADI) Model, 1997 [73]	USA	✓	✓			
Elder Abuse Network Training Manual, 2005 [74]	CAN	✓	✓		✓	
Elder Abuse Prevention, 2010 [75]	USA	✓				
Elder Abuse Resources Manual, 2000 [76]	CAN		✓			
Elder Abuse, Neglect, and Family Violence: A Guide for Health Professionals, 2009 [34]	USA	✓				
Elder Abuse: Assessment and Intervention Reference Guide, 2010 [77]	CAN				✓	✓
Elder abuse: Using Clinical Tools to Identify Clues of Mistreatment, 2000 [78]	USA	✓				
Elder Abuse: What to Look For, How to Intervene, 1997 [79]	USA	✓	✓			
Elder Assessment Instrument, 2003 [80]	USA	✓				
Elder Mistreatment Identification and Assessment, 2003 [81]	USA	✓				
Family Violence and Intervention Guidelines: Elder Abuse and Neglect, 2006 [28]	NZ	✓				
Financial Abuse Specialist Team Practice Guide, 2010 [82] [83]	USA		✓	✓	✓	✓
Forensic Nursing Files: Sexual Abuse of Older Adults, 2005 [84]	USA	✓				
Guidelines for Developing Elder Abuse Protocols: A South West Ontario Approach, 2011 [85]	CAN	✓	✓	✓	✓	✓
Guidelines for Intervention in Elder Abuse, 1996 [86]	UK	✓				
Identifying and Responding to Elder and Dependent Adult Abuse in Health Care Settings: Guidelines for California Health Care Professionals, no date [87] [88]	USA	✓	✓		✓	✓
Illinois Statewide Elder Abuse Social Service Program, 1996 [89]	USA		✓			
Improving Intervention in Intimate Partner Violence against Older Women: Guidelines for Social Services, 2013 [90]	PT		✓		✓	
In Hand: An Ethical Decision Making Framework, 2010 [91]	CAN	✓	✓		✓	✓
Intimate Partner Violence against Older Women: Contributions to the Manual on Policing Domestic Violence, 2013 [92]	PT		✓		✓	✓
Kentucky Medical Association: Abuse of Vulnerable Adults, no date [93]	USA	✓				
Learn how to Assess the Visible and Invisible Indicators and What to Do if you Recognize Abuse in an Older Patient, 2000 [94]	USA	✓				
Looking Beyond the Hurt: Service Provider's Guide to Elder Abuse, 2013 [36]	CAN	✓	✓	✓	✓	✓
Medical Assessment of Elder Abuse, 2004 [31]	USA	✓				
Mistreated and Neglected Elders: Social Work Assessment Intervention, Assessment Guide, 2006 [95]	USA	✓				
Ontario Network for the Prevention of Elder Abuse: Free From Harm Guide, no date [96]	CAN		✓			
PAHO Manual Part II: Abuse (Mistreatment) and Neglect (Abandonment) Diagnostic and Management Guide I, no date [97]	USA	✓	✓			
Principles of Assessment and Management of Elder Abuse, 2006 [98]	USA	✓	✓			
Procedural Guidelines for Handling Elder Abuse Cases, 2006 [32]	HK	✓	✓		✓	✓
Protocol for Law Enforcement: Responding to Victims of Elder Abuse, Neglect, and Exploitation, 2011 [99]	USA				✓	✓
Protocol for Responding to Abuse of Older People Living at Home in the Community, 2011 [100]	AUS	✓	✓			
Quick Reference to Adult and Older Adult Forensics: A Guide for Nurses and Other Health Care Professionals, 2010 [101]	USA	✓				
Regional Capacity Assessment Team (RCAT) Tool, 2008 [35]	CAN	✓	✓			
Risk Factors and Cutaneous Signs of Elder Mistreatment for the Dermatologist, 2013 [102]	USA	✓				
Safety Planning for Older Persons, no date [103]	CAN		✓			
Screening Tools and Referral Protocol for Stopping Abuse Against Older Ohioans: A Guide for Service Providers, 2001 [104]	USA		✓			
Sexual Violence in Later Life: A Technical Assistance Guide for Health care Providers, 2013 [105]	USA	✓				
Technical Assistance Manual for Older Adult Protective Services, 2007 [106]	USA	✓	✓	✓	✓	✓
The Health Care Provider's Reference Guide to Partner and Elder Abuse, 2007 [107]	USA	✓				
The Occupational Therapy Elder Abuse Checklist, 2001 [108]	USA	✓				
The Primary Care of Elder Mistreatment, 1999 [109]	USA	✓				
The Role of the Dentist in Recognizing Elder Abuse, 2008 [110]	CAN	✓				
Vulnerable Adults: The Prevention, Recognition and Management of Abuse, 2007 [111]	UK	✓				
Waterloo Region Committee on Elder Abuse: A Guide for those Working with Older Adults, 2008 [112]	CAN	✓	✓			✓
Victorian Government Practice Guidelines for Health Services and Community Agencies for the Prevention of Elder Abuse, 2009 [113]	AUS	✓	✓	✓	✓	✓

* AUS = Australia, CAN = Canada, HK = Hong Kong, NZ = New Zealand, PT = Portugal, UK = United Kingdom, USA = United States of America.

**H = Health, C/S = Community/Social Service, F = Finance, LE = Law Enforcement, and L = Legal.

More than four-fifths (81%) of responses identified in our review were developed with input from two or more professional groups or sectors. Knowledge users, those working in the sectors targeted, were involved in the development of most (85%) of the responses examined; these professionals were most commonly health care providers (59%), legal experts (19%), and law enforcement personnel (18%). Researchers/academics were involved in the development of 56% of the responses, followed by policy makers (38%), and public representatives (12%) (see Table 2).

**Table 2:** Development of the Responses to Elder Abuse.

	n	%
Stakeholder groups involved*		
Researcher/academic	38	56%
Policy maker	26	38%
Knowledge user	58	85%
Public representative	8	12%
**Number of methods used**		
No report of methods used	19	28%
One method only	33	49%
Two methods	13	19%
Three methods	3	4%
**Multiple methods used**	16	23%
Type of methods used*		
Previous guidelines, protocols, or related materials	42	62%
Consensus methods**	11	16%
Nonsystematic literature search	9	13%
Chart review	4	6%
Systematic literature search	1	1%
Interview/focus group	1	1%

*Categories are not mutually exclusive.

**No response documented having used a formal Delphi consensus survey.

Fewer than three-quarters (72%) of the responses examined described methods of development used; 23% listed more than one method. The most common method cited was use of pre-existing guidelines/protocols (62%). Consensus methods (e.g., consensus meetings, advisory groups) were used to inform 16%, and non-systematic literature reviews 13%, of responses (see Table 2).

Approximately, one third (35%) of responses reported having been validated in some capacity. Most commonly this included having been reviewed by external stakeholders and revised based on feedback before finalization (15%). Several responses had been pilot tested (10%) and/or evaluated (9%). For example, it was noted in *Procedural Guidelines for Handling Elder Abuse Cases* that

[T]he [Hong Kong Christian Service] … conducted a pilot run to test out the feasibility of the first draft of the Guidelines. … Drawing on the experience obtained from the pilot run, [it] made some amendments of the content of the draft Guidelines. Lastly, the Guidelines were further refined by the [Social Welfare Department] based on the views of members of the [Working Group on Elder Abuse]. [32]

and in *A Model Intervention for Elder Abuse and Dementia* that

[E]valuation involved assessment of the training program through participant completion of evaluation forms before training was initiated and after each session was completed. … critical review of agency protocols and analysis of client outcomes. … anecdotal reports [from staff] regarding cross-referrals and consultations following the training.” [33](pp. 495, 496)

Some (13%) responses had been endorsed by external organizations such as *Elder Abuse*, *Neglect*, *and Family Violence*: *A Guide for Health care Professionals*, endorsed by the Wisconsin Medical Society [34] (see Table 3).

**Table 3:** Validation of the Reponses to Elder Abuse.

	n	%
**Number of types of validation used**		
No report of validation	44	65%
One type only	17	25%
Two types	5	7%
Three types	1	1%
Four types	1	1%
**Multiple types of validation used**	7	10%
Type of validation used*		
Reviewed by external stakeholder	10	15%
Pilot tested	7	10%
Evaluated	6	9%
Plans to evaluate	2	3%
Endorsed by external organizations	9	13%

*Categories are not mutually exclusive.

### Recommendations relevant to a comprehensive hospital-based elder abuse intervention

Of the 1649 recommendations for potential implementation by a multidisciplinary intersectoral team of professionals in a comprehensive hospital-based elder abuse intervention extracted and collated, 149 were retained following the final relevancy review, and were coded and categorized into five themes: Initial contact (e.g., “Determine the level and urgency of safety concerns” [28]; n = 7); Capacity and consent (e.g., “[Determine the] client's perspective on the questions raised about their capacity” [35]; n = 8); Interview with older adult, suspected abuser, caregiver and/or other relevant contacts (e.g., “Assess longstanding relationship problems [dynamics] between victim and perpetrator” [29]; n = 69); Assessment: physical/forensic, mental, psychosocial, and environmental/functional (e.g., “Identify and document details of the neglect [as reported] (frequency, what needs aren't being met, etc.)” [36]; n = 41); and Care plan (e.g., “All [relevant] professionals should attend [multidisciplinary care committee meetings] wherever possible to assist the formulation of a welfare plan for the abused elder” [32]; n = 24) (see Table 4).

**Table 4:** Example Recommendations Relevant to a Comprehensive Hospital-based Intervention.

**Initial Contact**
“[Determine if] interpreter or [c]ultural [a]dvisor required.” [54]
“Determine the level and urgency of safety concerns.” [28]
“Determine if perpetrator still has access to the victim.” [106]
“Identify risk that is life threatening, including risk of homicide.” [28]
“Identify risk of suicide and self-harm.” [28]
“[Record] last name, first name, street address…telephone (home, work), age, date of birth, gender, [and] ethnicity.” [87]
“[Where sexual assault is suspected], encourage the victim to preserve evidence by not changing clothes, washing, using bathroom, drinking anything, combing hair or disturbing scene.” [106]
**Capacity and Consent**
“[Determine if] there [has] been a previous medical opinion that the client lacks capacity.” [35]
“[Determine] (1) whether mental deficits exist; (2) whether mental deficits significantly affect legal mental capacity; (3) a diagnosis; (4) whether a mental disorder is treatable; and (5) whether the mental deficits may be reversible.” [87]
“[Assess] memory (delayed recall of three items and response to questions related to temporal orientation); language (naming common objects, repeating a linguistically difficult phrase, following a three step command, and writing a sentence); spatial ability (copying a two-dimensional figure); and set-shifting (performing serial sevens or spelling the word “world” backwards).” [87]
“[Determine the] client's perspective on the questions raised about their capacity.” [35]
“If the person is able to understand and accept the consequences of decisions… [and there is] no consent [to care]: provide information, document abuse, and follow up plan to obtain consent (e.g. provide support, education).” [112]
“If the person is [not] able to understand and accept the consequences of decisions, contact substitute decision maker (SDM). If SDM is abuser or no SDM appointed, contact the public guardian and trustee’s office to investigate.” [112]
“Does victim appear to have capacity and ability to protect himself/herself? [If no i]nitiate process for [public guardian and trustee] or [f]amily/[f]riend petition for private Conservatorship.” [82]
“[Where the older adult lacks capacity]: If the elder has no relatives/guardian or the elder’s relatives/guardian refuse to allow him/her to receive the treatment, in the interest of the elder’s personal safety, the [healthcare provider] in charge should apply for the elder an emergency guardianship order so that the elder can be provided with the required medical services.” [32]
**Interview Older Adult, Suspected Abuser, Caregiver, and/or Other Relevant Contacts**
**Interview with Older Adult**
“[Keep w]hatever information a person chooses to share or whatever information becomes known about them … confidential except in specific situations, as dictated by law.” [29]
“Record the name(s), addresses, and telephone numbers of current or prior health care providers who have participated in caring for the patient in the past." [87]
“Record current use of medication(s) such as aspirin, nonsteroidal anti-inflammatory drugs, and/or [anti-coagulants] that the patient has been taking." [87]
“[Record c]oping: (a) wellness and disease management (e.g. diet, exercise, management of chronic conditions), (b) Coping styles and techniques, … (c) Use of psychotropic medications, history of psychiatric care/hospitalization, (d) History of non-functional coping approaches/behaviours (e.g. self-harm, hoarding, rituals, ruminating), (e) Use of alcohol/drugs (frequency, amount, any problems associated with use), (f) Sleeping patterns, (g) Alternative/traditional health practices.” [35]
“Ask the client about his or her expectations regarding care.” [73]
“Assess caregiving and social support.” [98]
“[Ask w]hat thoughts do you have about how your illness or care might affect others in your life?” [35]
“Assess longstanding relationship problems [dynamics] between victim and perpetrator.” [29]
“[Determine r]isk of abuse: (a) Risk factors/indicators (b) Nature of concerns (c) Client insight into any issues (d) Client’s ability to protect self from any mistreatment (i.e. degree of vulnerability) (e) Client report of safety and necessary care.” [35]
“Ask client about role expectations for self and caregiver.” [73]
“Try to assess whether the person "understands" and "appreciates" what is happening and what their needs are.” [70]
“Ask directly about abuse—‘We ask everyone about abuse in their lives because it is a concern for many people. Is there any person, or place in your life that makes you feel unsafe?’” [35]
“Document details of abuse [as reported] (type, frequency, and severity).” [36]
“Once the older victim begins to disclose information, ask the victim to describe the situation or incident in their own words.” [106]
“Provide best known time frame [for occurrence of abuse] (e.g., 2 days, 1 week, or ongoing).” [87]
“[Ask w]hat religious beliefs, past experiences, attitudes about social service agencies or law enforcement, or social stigmas may affect [older adult, caregiver, etc.] decisions to accept or refuse help from outsiders?” [29]
“With immigrant older adults, [ask] when did they come to [the country] and under what circumstances? Did they come alone or with family members? Did other family members sponsor them and, if so, what resources did those family members agree to provide? What is their legal status?” [29]
“Because it is common for more than one type of elder abuse to be taking place, be alert for signs and symptoms for all types of abuse and neglect.” [28]
***Specific questions*: *Financial Abuse***
“[Ask d]o you know your income and its sources?” [30]
“[Ask d]o you have a Power of Attorney?” [75]
“[Ask q]uestions about theft or improper control of money or property.” [20]
“[Ask h]ow do you get to the bank?” [30]
“[Ask d]o you have any assets?” [30]
“[Ask d]o you have any debt?” [30]
“[Ask w]ho does your finances?” [36]
“[Ask a]re you comfortable with how [the person who does your finances] handle[s] your finances?” [36]
“[Ask d]o you ever run out of money for food or worry about your rent?” [30]
“[Ask d]oes your family/friend come to you for money?” [30]
“[Ask d]oes anyone ever take anything from you or use your money without permission? Can you give me an example?” [36]
“[Ask h]ave you ever been asked to sign papers that you didn't understand?” [36]
***Specific questions*: *Neglect***
“[Ask t]ell me about your living situation. Are you happy with it?” [36]
“[Ask a]re you alone a lot?” [36]
“[Ask a]re you getting all the help that you need?” [36]
“[Ask d]oes anyone ever tell you that you're sick when you know you aren't?” [36]
“[Ask d]o you feel that your food, clothing, and medications are available to you at all times?” [36]
“[Ask w]hen was the last time you [were able] to see relatives and/or friends?” [36]
“[Ask h]as anyone ever failed [or refused] to help you when you were unable to help yourself?” [69]
“Ask directly if the patient has experienced being left alone, tied to chair or bed, or left locked in a room.” [73]
***Specific questions*: *Physical Abuse***
“[Ask h]as anyone ever hit, slapped, restrained or hurt you?” [59]
“[Ask h]ow did the person hurt you?” [71]
“[Ask w]hat part of your body was hurt?” [71]
***Specific questions*: *Psychological Abuse***
“[Ask d]o you sometimes feel nervous or afraid?” [29]
“[Ask d]oes anyone call you names or insult you?” [29]
“[Ask a]re you able to freely communicate with your friends and/or other family members?” [29]
“[Ask a]re you often yelled at by someone? Who? What do they say?” [29]
“[Ask d]oes anyone threaten or intimidate you? Who? What do they say or do?” [29]
“[Ask w]ho makes decisions about your life, such as how or where you will live?” [29]
“[Ask h]as anyone ever threatened to send you to a nursing home?” [29]
“[Ask h]as anyone ever threatened to send you back home (i.e. country of origin)?” [29]
“[Ask d]oes anyone ever tell you that you are no good?” [29]
“Assess if patient senses being ignored or is made to feel like a burden in any way.” [73]
***Specific questions*: *Sexual Abuse***
“[Ask d]oes anyone make lewd or offensive comments to you?” [29]
“[Ask d]oes anyone approach you in a way that causes you to feel uncomfortable?” [29]
“[Ask d]oes anyone touch you without your consent?” [71]
“[Ask d]oes anyone touch you sexually without your consent?” [71]
“[Ask d]oes someone make you touch him/her in a sexual way without your consent?” [71]
“[Ask d]oes someone force you into having sex without consent?” [71]
**Interview Suspected Abuser, Caregiver, and/or Other Relevant Contacts**
“[Record] last name, first name, street address…telephone (home, work), age, date of birth, gender, ethnicity, [and] relationship to the older adult.” [87]
“Assess if the caregiver understands the older adult’s needs and prognoses.” [87]
“Assess whether the caregiver is experiencing stress related to the older adult or other circumstances.” [87]
“Assess whether the caregiver has sufficient emotional, financial, and intellectual ability to carry out care giving tasks.” [87]
“[Determine] carer’s understanding of patient’s illness (care, needs, prognosis, and so on).” [20]
“[Gather] explanations for injuries or physical findings” [20] “[For example, y] our mother[/father, etc.] is suffering from malnourishment and/or dehydration. [Ask h]ow do you think she got this way?” [73]
“[Ask h]ow do you cope with having to care for your mother[/father, etc.] all the time?” [73]
“Determine willingness for intervention.” [95]
“Assess the suspected perpetrator’s degree of dependence on the elder’s income, pensions, or assets?” [73]
“Pay particular attention to any discrepancies and inconsistencies in the accounts of abuse obtained from the older woman, the alleged abuser, and other information sources.” [96]
“Make collateral contact promptly, before caregiver attempts to collude with them.” [73]
**Assessment: Physical/Forensic, Mental, Psychosocial, and Environmental/Functional**
“In cases where forensic evidence has been collected, provide to the police with patient/substitute decision maker consent.” [30]
**Physical/Forensic Assessment**
“[Record h]eight, [w]eight, [p]rior [w]eight, [d]ate of [p]rior [w]eight." [87]
“Record vital signs to include postural pulse and blood pressure." [87]
"Evaluate sensory abilities." [72]
“[E]valuate abused elders for evidence of infection, dehydration, electrolyte abnormalities, malnutrition, improper medication administration, and substance abuse." [101]
“Create a chronological history of recorded [visits] to the emergency, incidences from the chart together with anecdotal information from other sources to clarify the picture." [74]
“Conduct a general physical exam and record findings.” [87]
“[Conduct g]ynecologic procedures to rule out [a sexually transmitted infection] by sexual assault." [73]
“Be observant for erythema (redness), abrasions, bruises, swelling, lacerations, fractures, bites, pressure ulcers, cachexia or evidence of dehydration, and burns.” [87]
“Document … pain." [106]
“[D]ocument circumstances [of injury] (e.g., client was pushed, client has balance problem, patient was drowsy from medications and fell)." [73]
“Photograph injuries and other findings according to local policy using proper photographic techniques." [87]
“Arrange … to have follow-up photographs taken in 1–2 days after the bruising develops more fully." [87]
“[Document c]ircumscribed nuchal rope burns or hand imprints [which] indicate recent strangulation attempts or bondage." [78]
“Document whether or not a voice recording of strangulation injuries was made." [87]
“[Collect] the victim's clothes, bed sheets and any other possible evidence." [106]
“Collect foreign materials such as fibers, sand, hair, grass, soil, and vegetation." [87]
“Collect biological samples for testing from victims.” [72]
“[Order l]aboratory tests … [to] confirm … or exclude …physical abuse includ[ing] hematuria, myoglobinuria, elevated serum creatine phosphokinase, lactate dehydrogenase, erythrocyte sedimentation rates, microscopic hair analysis, coagulation times, bone scans or x-rays, and CT and MRI." [78]
**Mental Health Assessment**
“[Ask about h]istory of depression, anxiety, PTSD, suicide risk. . . delusions and hallucinations. " [87]
“Describe the patient’s general demeanor/behavior during exam." [87]
“Assess for: changes from previous level in mental status and neurological exam." [73]
“[Perform n]europhysical testing … if the client's [initial] mental status exam shows incapacity” [73]
“[Assess] basic skills for financial management (e.g., unable to write a check, count change, complete simple calculations, etc.)." [67]
**Psychosocial Assessment**
“[Record c]urrent living situation…housing and co-residents." [35]
“[Record] social and family history: (a) Family of origin / (b) Education (formal, informal meaning to the client), (c) Occupation, (d) Work skills …, Hobbies/interests … (k) Social groups (e.g. church/faith community, senior group, etc.)." [35]
“Find … out how the client spends a typical day … to determine the degree of dependence on others and to find out who the client's most frequent and significant contacts are." [73]
“[Ask w]hat role do older adults play in the family? In the community?" [29]
“[Ask w]ho makes decisions about how family resources are used? About other aspects of family life?" [29]
“[Ask w]ho, within the family, do members turn to in times of conflict?" [29]
“[Ask w]ho, within the family, is expected to provide care to frail members? What happens when they fail [or refuse] to do so?" [29]
“Have the client report any recent crises in family life." [73]
“Determine the importance of spirituality to the elder." [29]
**Environmental/Functional Assessment**
“Describe the patient’s general physical appearance and hygiene." [87]
“Describe condition of patient’s glasses, dentures, hearing aids, wheelchairs, canes, walkers, etc." [87]
“Does client [have] enough clothes?" [67]
“Ask about any pets, and what the pets need, as this is often an important consideration in making decisions about staying or leaving." [34]
“Assess the client's ability to perform activities of daily living. … Basic living skills that require assessment are the clients' ability to groom themselves, to dress, to walk, to bathe, to use the toilet, and to feed themselves." [73]
“Indicate any limitations [in] functional history." [87]
“[Record] Coping style and techniques—Ask the client: What lessons have you learned about how to cope with life from day to day? Are there ways you wish you cope better?" [35]
“[Determine] who is the designated carer if [independence with activities of daily living] are impaired." [20]
“Identify and document details of the neglect according to the senior (frequency, what needs aren't being met, etc.)" [36]
**Care Plan**
“Assign a case manager.” [32]
“Address immediate basic needs such as clothing, transportation (cab fare or transit tokens), food and shelter first.” [55]
“Arrange for the provision of supportive services including … temporary medications, assistive devices.” [72]
“[Arrange] short hospital stay or repeated contact for further assessment and case planning.” [73]
“If a client reveals information that must be reported… work to include the client in the reporting process.” [57]
“If the older person is at serious risk, [invoke] an interim order to allow the older person to be removed to alternative accommodation.” [100]
“Find a safe place, such as a shelter, a hospital, a home of a trusted friend or family member or emergency placement in a long term care facility or retirement home.” [29]
“Educate the patient to recognize and use community resources such as emergency shelter, elder shelter, transportation, police intervention, and legal action.” [93]
“Refer … patient, family members, or both to appropriate services (eg, social work, counselling services, legal assistance, and advocacy.” [20]
“Ask whether they have a means of getting to the services you have recommended or referred to them; and offer help if required.” [55]
“Provide information to the older person about the following: That what is happening is not their fault; that many older people experience this mistreatment by family members; and that there are people who can them find ways to stop the mistreatment / That abuse escalates over time and without some kind of actions it’s unlikely to stop / That safety planning is necessary to keep them safe when the abuse happens again.” [29]
“Develop and review safety plan.” [20]/ “Teach your older patients … safeguards to help them avoid abusive situations. Stay sociable… Stay active… Stay organized… Stay informed.” [94] / “Explain to the patient that anticipated high-risk times can be reduced by having family members, friends, and other support system members visit during those times or periods of time, or by participating in community activities and agency programs, such as senior center, an adult daycare, church, and so forth.” [93]
“Where abuse is related to caregiver stresses, [take] actions … to reduce these factors: respite/home care to reduce caregiver burden for high priority clients, supportive therapy or medical intervention for caregiver, education.” [74]
“When an Adult Declines the Care Plan: Consider the reasons why the support and assistance was declined / Coordinate the supports and assistance that will be accepted / Reassess the level of risk to the adult and assets / … / Consider using legal tools to protect the adult/assets / Consider using emergency provisions to protect the adult/assets / Put the recommended care plan and rationale in writing, and give to the person responsible for implementation/document the reasons why the care plan was declined / Have a clear plan for following up and monitoring the situation.” [30]
“All [relevant] professionals should attend [multidisciplinary care committee meetings] wherever possible to assist the formulation of a welfare plan for the abused elder.” [32]
“[Invite t]he elder/family members/guardians/suspected abuser … to attend the entire [or] part of the [multidisciplinary care committee meeting] … after the initial recommendations on the welfare plan have been made.” [32]
“[P]repare a brief report for the case and submit it to the participating professionals before the [multidisciplinary care committee meeting].” [32]
“[E]stablish clear expectations to the [multidisciplinary care committee] regarding what observations should be communicated back to the Case Manager for further actions” [76]
“[M]aintain contact with all [multidisciplinary care committee] members to ensure a smooth implementation of the welfare plan.” [32]
“[N]otify and consult all members on the drastic changes in the elder’s situation. A review conference may also be considered where necessary.” [32]
“Maintain an ongoing telephone or in-person contact [with older adult] to further assess the situation, to diminish the fear and anxiety of the vulnerable person and to establish a trusting relationship.” [76]
“Attempt to engage other friends, neighbours or relatives to support the person, providing the individual consents.” [76]
“[R]eview and update the safety plan at regular intervals” [96]
“[T]erminate [the case] … when any of the following circumstances occur: When requested by the adult … / The adult no longer needs … services / The adult leaves the … area of jurisdiction… / The adult dies.” [59]

Note: The same/similar recommendations may have been made in multiple documents, however, a direct quotation from a single representative citation is provided for each. Each recommendation would only be applied where relevant, appropriate, and with consent (where required).

## Discussion

The prevalence, negative sequelae, lack of available services, and increasing aging population globally indicate a strong need for effective comprehensive health service interventions to address elder abuse. Our systematic scoping review of the grey and scholarly literatures identified 68 elder abuse guidelines, protocols, and related materials with recommendations relevant to a multidisciplinary intersectoral hospital-based intervention. The recommendations possibly pertinent to forensic nurse examiner models of care focused on initial contact with the older adult, assessing the older adult’s mental capacity and obtaining informed consent, interviewing the older adult, suspected abuser, caregiver, and/or other relevant contacts, providing physical/forensic, psychological, environmental/functional assessments, and formulating and delivering a care plan. These recommendations, upon further evaluation and with proper training and organizational supports, could be implemented within existing forensic nurse examiner programs [24].

Although elder abuse is a problem that has been documented worldwide [37], our review revealed that more than 4-in-5 responses relevant to hospital-based care were developed in the United States or Canada and, therefore, may not be entirely applicable, to other jurisdictions. This may be because the multiple databases searched tend to retrieve results from North America and Europe [38]. Additionally, the limitation of our review to inclusion of English language documents only may have restricted our ability to capture the full range of relevant international responses. The health sector and the community/social service sector were most often the target audience of responses. Only a handful of documents were aimed at those working in the financial sector, which may be problematic given that some population-based studies have shown that financial/material abuse is one of the most common types of elder abuse experienced [39–42].

In this review, representatives from the public were identified as underrepresented in the development of responses to elder abuse—involved in the construction of just 12% of the reviewed responses. This is similar to findings from another review article [43], and contrary to recommendations for developing guidelines [44,45]. As the responses examined are designed explicitly to address the needs of older adults where abuse is suspected and or has occurred, it is critical to ensure that their first hand perspectives and experiences are considered in shaping services. This group of stakeholders should be better engaged in the development of future interventions.

A substantive proportion of the elder abuse responses reviewed did not report their methods of development, making it impossible to comment on their rigor. The overwhelming majority drew on recommendations from pre-existing materials that themselves were not evidence-based. This is consistent with a systematic review by Shaneyfelt et al. [46] who found that only 33.6% of the guidelines they reviewed adhered to the established methodological standards for the identification and summary of evidence. Only one response in our sample of 68 was developed using a systematic review of the literature. Eleven responses were based on findings from consensus methods, although none used a formal Delphi consensus survey, which allows for the integration of the opinions of many different experts, and has been used successfully in other areas of elder abuse research [47–49].

We found that in almost two thirds of elder abuse responses reviewed there was no report of validation. The most common form of validation documented, in 15% of cases, was external stakeholder review. Only 6 responses of 68 had been evaluated, suggesting a significant gap between development and implementation of recommendations. This fact may be a disservice to older adults, as thorough evaluation of interventions is critical to developing evidence informed responses to elder abuse that prevent harm. It has been previously demonstrated that rigorously developed and evaluated clinical guidelines do improve clinical practice when implemented [50].

This review has strengths and limitations. The broad search strategy used in this review is congruent with the complex and multifaceted nature of addressing the elder abuse problem and as such captured documents developed by a variety of important stakeholders. The resulting diverse sample of responses allows for the integration of perspectives from multiple disciplines and sectors in the development of a comprehensive hospital-based elder abuse intervention. That said, although every attempt was made to capture all relevant guidelines, protocols, and related materials, some may have been missed. For example, post search and analysis, we found an elder abuse guideline for occupational therapists, although upon examination, it contributed no additional relevant recommendations to a hospital-based response [51]. The inclusion of a range of document types made a formal quality assessment of the included responses unfeasible as there is no currently available validated tool for that purpose [44], although we did describe the methods used to develop and validate the responses. Given the paucity of high quality studies assessing elder abuse interventions, as cited in a previous systematic review [52], we were unable to systematically evaluate the strength of the evidence for individual recommendations. To address this lack of evidence to support the recommendations, a next step in the development of any hospital-based response to address elder abuse must be a further evaluation of the extracted recommendations.

### Future Research

The next phase of this research is a Delphi consensus survey to determine the final components of care in the intervention under development, in which the nurse examiner will work with other healthcare providers and collaborators from the community/social service, finance, law enforcement, and legal sectors to address the complex functional, medical, legal, and social, needs of abused older adults. A group of 33 experts in hospital-based violence programs have been assembled to review and rank the recommendations extracted in this review for their importance to a comprehensive hospital-based response. This type of program of research, which addresses a high priority area in the field of aging and a significant gap in health research, will lead to an intervention that could improve the quality of life of abused older women and men and prevent further victimization.

## Supporting Information

S1 Appendix PRISMA Checklist. (DOCX)

S2 Appendix Hospital-based Elder Abuse Intervention Systematic Scoping Review Search Strategy. (DOCX)

S1 Dataset Hospital-based Elder Abuse Intervention Systematic Scoping Review Dataset. (XLSX)
