# Supplementary material for: Development of a Comprehensive Hospital-Based Elder Abuse Intervention: An Initial Systematic Scoping Review
Source: PLoS One. 2015 May 4;10(5):e0125105. doi: 10.1371/journal.pone.0125105 (PMC4418829; doi:10.1371/journal.pone.0125105)
Supplement: S2 Appendix — (DOCX) [file pone.0125105.s002.docx]

**SCHOLARLY LITERATURE SEARCH**

**MEDLINE**

**January 1, 1995** **to October 01, 2013 (October Week 1 2013)**

1 Elder Abuse/ 1885

2 (elder abuse or elder neglect or elder mistreatment or elder maltreatment).mp. 2040

3 ((elderly or older or senior) adj3 (abuse or neglect or mistreatment or maltreatment)).mp. 669

4 1 or 2 or 3 2373

5 interven*.mp. 609850

6 response*.ti,ab. 1911460

7 guideline*.mp. 277540

8 protocol*.mp. 379323

9 consensus.mp. 115628

10 recommendation*.mp. 147789

11 evidence based.mp. 96111

12 best practice*.mp. 6111

13 social support.mp. 61060

14 mandatory reporting.mp. 2843

15 abuse reporting.ti,ab. 127

16 decision making.mp. 126041

17 forensic.mp. 49080

18 planning.mp. 242832

19 (policy or policies).mp. 217091

20 Health Services for the Aged/ 15012

21 Health Knowledge, Attitudes, Practice/ 73636

22 5 or 6 or 7 or 8 or 9 or 10 or 11 or 12 or 13 or 14 or 15 or 16 or 17 or 18 or 19 or 20 or 21 3717122

23 4 and 22 1069

24 limit 23 to yr="1995 -Current" 912

25 remove duplicates from 24 861

**EMBASE**

**January 1, 1995 to Oct 11, 2013 (2013 Week 40)**

1 Elder Abuse/ 486

2 (elder abuse or elder neglect or elder mistreatment or elder maltreatment).mp. 1383

3 ((elderly or older or senior) adj3 (abuse or neglect or mistreatment or maltreatment)).mp. 817

4 1 or 2 or 3 1957

5 interven*.mp. 741747

6 response*.ti,ab. 2074694

7 guideline*.mp. 388220

8 protocol*.mp. 368660

9 consensus.mp. 131528

10 recommendation*.mp. 183536

11 evidence based.mp. 136093

12 best practice*.mp. 8553

13 social support.mp. 63102

14 mandatory reporting.mp. 2629

15 abuse reporting.ti,ab. 144

16 decision making.mp. 249223

17 forensic.mp. 71085

18 planning.mp. 354119

19 (policy or policies).mp. 296796

20 Health Services for the Aged/ 34053

21 Health Knowledge, Attitudes, Practice/ 81663

22 5 or 6 or 7 or 8 or 9 or 10 or 11 or 12 or 13 or 14 or 15 or 16 or 17 or 18 or 19 or 20 or 21 4397282

23 4 and 22 1062

24 limit 23 to yr="1995 -Current" 927

25 remove duplicates from 24 904

**PsycINFO**

**January 1, 1995 to Oct 11, 2013 (October Week 2 2013)**

1 Elder Abuse/ 1174

2 (elder abuse or elder neglect or elder mistreatment or elder maltreatment).mp. 1388

3 ((elderly or older or senior) adj3 (abuse or neglect or mistreatment or maltreatment)).mp. 760

4 1 or 2 or 3 1808

5 interven*.mp. 235117

6 response*.ti,ab. 393549

7 guideline*.mp. 40908

8 protocol*.mp. 27633

9 consensus.mp. 17788

10 recommendation*.mp. 60657

11 evidence based.mp. 22609

12 social support.mp. 42323

13 decision making.mp. 73745

14 forensic.mp. 14583

15 planning.mp. 56075

16 (policy or policies).mp. 102727

17 abuse reporting.mp. 1003

18 mandatory reporting.mp. 237

19 exp Government Policy Making/ 31414

20 exp Health Knowledge/ or exp Health Attitudes/ 12209

21 best practice*.mp. 7779

22 5 or 6 or 7 or 8 or 9 or 10 or 11 or 12 or 13 or 14 or 15 or 16 or 17 or 18 or 19 or 20 or 21 936039

23 4 and 22 1005

24 limit 23 to yr="1995 -Current" 797

25 remove duplicates from 24 789

**Grey literature search**

Concluded on December 6, 2013

| **Organization Name** | **URL** |
| --- | --- |
| AARP — United States | <http://www.aarp.org/> |
| AARP International | http://www.aarpinternational.org/ |
| ACT - Elder Abuse Prevention, Information and Referral Line | http://www.communityservices.act.gov.au/wac/ageing/elder_abuse_prevention__and__assistance |
| Action On Elder Abuse | http://www.elderabuse.org.uk/ |
| Administration On Ageing | http://www.aoa.gov/ |
| Administration on Aging, Department of Health and Human Services | <http://www.aoa.gov/> |
| Advocacy Centre for the Elderly | http://www.advocacycentreelderly.org/index.php |
| Age & Opportunity — Ireland | http://olderinireland.ie/ |
| Age Action Ireland — Ireland | http://www.ageaction.ie/ |
| Age Concern - New Zealand | http://www.ageconcern.org.nz/ |
| Age Platform Europe | <http://www.age-platform.org/> |
| Age UK | http://www.ageuk.org.uk/ |
| Age-in-Action — The South Africa Council for the Aged | <http://www.age-in-action.co.za/new/> |
| Age-related Macular Degeneration Alliance International | http://www.amdalliance.org/home.html |
| Aged Care Maldives | <http://agedcaremaldives.org/index.php?pagename=home> |
| Ageless Alliance | http://www.agelessalliance.org/ |
| AgeSong — United States | http://www.agesong.com/index.php#sthash.w2yrJ9dm.dpbs |
| Alzheimer Society of Finland/ Muistiliitto — Finland | http://www.muistiliitto.fi/en/home |
| Alzheimer's Australia National | http://www.fightdementia.org.au/ |
| Alzheimer’s Association | http://www.alz.org/ |
| American Society on Aging (ASA) | http://www.asaging.org/ |
| ANBO – the Advocate for Seniors- Netherlands | http://www.anbo.nl/ |
| Area Agency on Aging | <http://www.eldercare.gov/> |
| Argentinian Federation of Gerontological Societies | http://www.gerontogeriatria.org/ |
| Association for Gerontology in Higher Education | <http://www.aghe.org/> |
| Association Québécoise de Gérontologie (AQG) | http://www.aqg-quebec.org/fr/accueil.aspx?sortcode=1 |
| Athashri Foundation | http://www.pscl.in/athashri |
| Atlantic Coordinating Committee on Crime Prevention and Community Safety | http://acc-cca.org/en/index.php/en/news/P6/ |
| Australia Network for Prevention of Elder Abuse | http://www.anpea.com.au/ |
| Australian Association of Gerontology | http://www.aag.asn.au/ |
| Australian National Health and Medical Research Council | <http://www.nhmrc.gov.au/> |
| Bangladesh Gerontological Association | http://www.bgabd.com/event/event.php |
| Bank of Montreal | http://www.bmo.com/main/personal?nav=top |
| Barbados Association of Retired Persons (BARP) | http://www.barpbb.com/ |
| Baycrest Health Sciences | http://www.baycrest.org/ |
| British Columbia Centre for Elder Advocacy and Support (Formerly BC Coalition to Eliminate Abuse of Seniors) | http://bcceas.ca/ |
| Brotherhood of St Laurence | http://www.bsl.org.au/ |
| Canadian Association of Occupational Therapists | [http://www.caot.ca](http://www.caot.ca/) |
| Canadian Association of Retired Teachers | http://www.acer-cart.org/CNSOwebpage/CNSOinfo.htm |
| Canadian Association on Gerontology | http://cagacg.ca/ |
| Canadian Elder Law | http://www.canadianelderlaw.ca/ |
| Canadian Medical Association | https://www.cma.ca/En/Pages/clinical-practice-guidelines.aspx |
| Canadian Network for the Prevention of Elder Abuse | http://cnpea.ca/ |
| Caregiver Homes | http://www.caregiverhomes.com/ |
| Carers Victoria | http://www.carersvictoria.org.au/ |
| Catholic Social Services | http://www.catholicsocialservices.ab.ca/CatholicSocialServices/default.aspx |
| Center for Disease Control and Prevention | <http://www.cdc.gov/violenceprevention/elderabuse/index.html> |
| Center for Elders and the Courts | http://www.eldersandcourts.org/ |
| Center on Elder Abuse and Neglect - University of California | http://www.centeronelderabuse.org/ |
| Central Union for the Welfare of the Aged/ Vanhunstyon Keskusliitto — Finland | http://www.vtkl.fi/fin/in_english/ |
| Central Welfare Council Peninsular Malaysia | http://www.mpksm.org.my/v1/index.php |
| Centrale Samenwerkende Ouderenorganisaties — Netherlands | http://www.site-supply.nl/cso/ |
| Centre for Policy on Ageing — United Kingdom | http://www.cpa.org.uk/index.html |
| Centre for Research and study of Aging — Israel | http://hw2.haifa.ac.il/index.php/he/researchcenters/60-gerontology/gero-general/23-study-of-aiging |
| Centre of Excellence for Alzheimer's Disease Research — Australia | http://www.ecu.edu.au/ |
| Clube Piratininga — Brazil | http://www.clubepiratininga.org.br/ |
| Community and Aid Sponsorship Program (CASP) — India | http://www.caspindia.org/webc/index.php |
| Community Development Volunteers for Technical Assistance Cameroon | http://www.cdvtacameroon.org/ |
| Community Legal Education Ontario | http://www.cleo.on.ca |
| Confederación Española de Aulas de la Tercera Edad (CEATE) — Spain | http://ceate.es/ |
| Confederación Española de Organizaciones de Mayores (CEOMA) — Spain | http://www.ceoma.org/es/ |
| COTA Australia for Older Australians | http://www.cota.org.au/ |
| Council of Europe | http://hub.coe.int/web/coe-portal |
| DaneAge Association (Ældre Sagen) | http://www.aeldresagen.dk/om-os/in-english/Sider/default.aspx |
| Dementia South Africa | http://www.dementiasa.org/ |
| Department of Health and Ageing — Australia | http://www.health.gov.au/ |
| Department of Social Development — South Africa | http://www.dsd.gov.za/ |
| Dobroe Delo (RPFAE) — Russia | http://www.dobroedelo.ru/ |
| Economic and Social Commission for Asia and the Pacific | http://www.unescap.org/ |
| Edmonton Seniors Coordinating Council | http://www.seniorscouncil.net/ |
| Elder Abuse Prevention Unit | [http://www.eapu.com.au](http://www.eapu.com.au/) |
| Elder Financial Protection Network (EFPN) | http://www.elderfinancialprotection.org/ |
| Elder Maltreatment Alliance | [http://www.eldermaltreatment.com](http://www.eldermaltreatment.com/) |
| Elder Rights Advocacy (ERA) - an aged care rights and advocacy service for older people (or their representatives) | http://www.era.asn.au/ |
| Elderly Commission — Hong Kong | http://www.elderlycommission.gov.hk/ |
| ETZA Self Help - Isreal | http://self-help.sheatufim.org.il/index-eng.php |
| EURAG Austria — Austria | http://www.eurag.at/english%20information.htm |
| Family Caregiver Alliance | <http://www.caregiver.org/> |
| Family Violence and the Workplace | http://www.toolkitnb.ca/ |
| Federación de Pensionistas y Jubilados de CCOO — Spain | http://www.pensionistas.ccoo.es/webpensionistas/ |
| Federation of Patients and Consumer Organisations in the Netherlands (NPCF) | http://www.npcf.nl/index.php?option=com_content&view=article&id=4164&Itemid=31 |
| Feros Care — Australia | http://www.feroscare.com.au/ |
| Foundation for Older Persons' Development (FOPDEV) — Thailand | http://fopdev.or.th/ |
| Friends of the International Federation on Aging (FOIFA) — Japan | http://www.foifa.or.jp/ |
| Full Circle of Care | http://www.fullcirclecare.org/ |
| German Centre of Gerontology (Deutsches Zentrum für Altersfragen) | http://www.dza.de/en/ |
| German National Association of Senior Citizens Organisations (BAGSO) | http://www.bagso.de/ |
| Gero Web - Wayne State University Institute of Gerontology | http://www.iog.wayne.edu |
| Geron Foundation — Romania | http://www.geron.ro/ |
| Gerontological Society of America | [http://www.geron.org](http://www.geron.org/) |
| Gesellschaft für Aktives Altern und Solidarität der Generationen | http://www.generationen.at/ |
| Global Action on Aging (GAA) | http://www.globalaging.org/ |
| Government of New Brunswick | http://www2.gnb.ca/ |
| Grantmakers in Aging | http://www.giaging.org/ |
| Grüne SeniorInnen — Austria | http://seniorinnen.gruene.at/index.php |
| Guidelines | http://www.guidelines.co.uk/ |
| Guidelines International Network | http://www.g-i-n.net/ |
| Haldimand and Norfolk Community Response Network | http://www.hncrn.ca/links.html |
| Harmony for Silvers Foundation — India | http://www.harmonyindia.org/ |
| Hawkesbury Living — Australia | http://www.hawkesburyliving.com.au/ |
| Healing Journey | http://www.thehealingjourney.ca/ |
| Helen Hamlyn Centre for Design — United Kingdom | http://www.rca.ac.uk/research-innovation/helen-hamlyn-centre/ |
| Hellas 50+ — Greece | http://www.50plus.gr/ |
| HelpAge India — India | http://www.helpageindia.org/ |
| HelpAge International | http://www.helpage.org/ |
| Heritage Hospital — India | http://www.heritagehealthcareindia.com/index.aspx |
| Home Instead Senior Care — United States | http://www.homeinstead.com/ |
| Hong Kong Council of Social Service | http://www.hkcss.org.hk/e/ |
| Hong Kong Society for the Aged (SAGE) | http://www.sage.org.hk/ |
| Human Echo, Affairs and Values of Eastern Nations (HEAVEN) — Pakistan | http://www.heavenpak.org/ |
| Independent Age — United Kingdom | http://www.independentage.org/ |
| Institute for Human Development, Life Course and Aging - University of Toronto | <http://aging.utoronto.ca/> |
| Institute for the Prevention of Crime website | <http://www.sciencessociales.uottawa.ca/ipc/eng/> |
| Institute of Older People and Social Services (IMSERSO) — Spain | http://www.imserso.es/imserso_01/index.htm |
| Institute on Violence, Abuse and Trauma (IVAT) | http://www.fvsai.org/ |
| International Association of Forensic Nurses | http://www.forensicnurses.org/? |
| International Association of Gerontology and Geriatrics | <http://www.iagg.info/> |
| International Association of Homes and Services for the Ageing (IAHSA) | http://www.iahsa.net/index.aspx |
| International Federation of the Red Cross | [www.ifrc.org](http://www.ifrc.org/) |
| International Federation on Aging | <http://www.ifa-fiv.org/?s=elder+abuse> |
| International Labour Organisation (ILO) | http://www.ilo.org/global/lang--en/index.htm |
| International Longevity Center - Japan | http://www.ilc-alliance.org/index.php/members/details/ilc-japan |
| International Longevity Centre Global Alliance (ICL) | http://www.ilc-alliance.org/ |
| International Network for the Prevention of Elder Abuse (INPEA) | http://www.inpea.net/ |
| International Network for the Prevention of Elder Abuse Japan | http://www.inpea.jp/about-2 |
| Intimate Partner Violence Against Older Women | http://www.ipvow.org/en/ |
| Istitute per lo Sviluppo della Formazione dei Lavoratori (ISFOL) — Italy | http://www.isfol.it/ |
| Janaseva Foundation — India | http://janasevafoundation.org/ |
| Justice Canada—Family Violence Initiative | http://www.justice.gc.ca/eng/cj-jp/fv-vf/ |
| Kentucky Medical Association | https://www.kyma.org/content.asp |
| Korean Association of Retired Persons — South Korea | http://www.karpkr.org/main/main.asp |
| KY Cabinet for Health & Family Services, Elder Abuse | http://chfs.ky.gov/dcbs/dpp/eaa/ |
| L'Agence Nationale D'Accréditation et D'Evaluation en Santé | <http://www.has-sante.fr/portail/jcms/fc_1249603/en/accreditation-certification> |
| Les Petits Frères des Pauvres — France | http://www.petitsfreres.asso.fr/ |
| Lutherwood — Canada | http://www.lutherwood.ca/ |
| Manitoba Network for the Prevention of Abuse of Older Adults | http://www.olderadultabuse.mb.ca/ |
| Matia Innova — Spain | http://www.matiainstituto.net/en |
| Matica Umirovljenika Hrvatske — Croatia | http://www.muh.hr/ |
| Mauritius Council of Social Service — Mauritius | http://www.macoss.intnet.mu/ |
| Meals on Wheels Japan — Japan | http://www.mow.jp/ |
| Medicare | [www.medicare.gov](http://www.medicare.gov/) |
| Ministry of Senior Citizens — Israel | http://vatikim.gov.il/ |
| Ministry of Social Security, National Solidarity and Reform Institutions — Mauritius | http://socialsecurity.gov.mu/English/Pages/default.aspx |
| Mission Armenia | http://www.mission.am/en |
| Monitoring RIS- Forum 50+ | http://www.monitoringris.org/index.php?id=204 |
| Mouvement des Ainés du Québec - La Fédération de l’Âge d’Or du Québec | http://www.fadoq.ca/ |
| Muriel McQueen Fergusson Center for Family Violence Research | http://www.unb.ca/ |
| National Ageing Research Institute (NARI) — Australia | http://www.mednwh.unimelb.edu.au/index.htm |
| National Center on Elder Abuse | <http://www.ncea.aoa.gov/> |
| National Centre for the Protection of Older People | http://www.ncpop.ie/ |
| National Clearinghouse on Abuse in Later Life | [www.ncall.us](http://www.ncall.us/) |
| National Committee for the Prevention of Elder Abuse | <http://www.preventelderabuse.org/> |
| National Council for the Elderly (NCOA) — United States | http://www.ncoa.org/ |
| National Criminal Justice Reference Service site (NCJRS) | https://www.ncjrs.gov/ |
| National Guardianship Association | <http://www.guardianship.org/> |
| National Guideline Clearinghouse | [http://www.guideline.gov](http://www.guideline.gov/) |
| National Indian Council on Aging | http://nicoa.org/ |
| National Initiative for the Care of the Elderly (NICE) | http://www.nicenet.ca/ |
| National Institute for Health and Care Excellence | <http://www.nice.org.uk/> |
| National Institute on Ageing | http://www.nia.nih.gov/ |
| National Online Resource Center on Violence Against Women | [www.vawnet.org](http://www.vawnet.org/) |
| National Resource Centre on LGBT Ageing | http://www.lgbtagingcenter.org/resources/index.cfm?s=5 |
| National Seniors Australia — Australia | http://www.nationalseniors.com.au/ |
| National Sexual Violence Resource Center | http://www.nsvrc.org/ |
| NB Silent Witness | [http://www.silentwitness.ca](http://www.silentwitness.ca/) |
| New South Wales - Elder Abuse Helpline | http://www.tars.com.au/ |
| New World Hope Organization (NWHO) — Pakistan | http://www.newworldhope.org/ |
| New Zealand Guidelines Group | <http://www.health.govt.nz/about-ministry/ministry-health-websites/new-zealand-guidelines-group> |
| NoAgeSite.com — France | http://www.noagesite.com/index.cgi/en |
| Norwegian Centre for Dementia Research | http://www.nordemens.no/ |
| Norwegian Elder Abuse | http://www.hioa.no/eng/ |
| Nyugdíjasklubok és Idosek "Életet az éveknek" Országos Szövetsége — Hungary | http://www.eletetazeveknek.hu/ |
| Office of the Public Advocate | http://www.publicadvocate.vic.gov.au/ |
| Older People's Commission of Wales — United Kingdom | http://www.olderpeoplewales.com/en/Home.aspx |
| Ontario Long Term Care Association — Canada | http://www.oltca.com/ |
| Ontario Network for the Prevention of Elder Abuse | http://www.onpea.org/ |
| Ontario Provincial Police - Seniors Assistance Team | http://www.opp.ca/ |
| Ontario Seniors' Secretariat | http://www.seniors.gov.on.ca/ |
| Österreichische Plattform für Interdisziplinäre Alternsfragen (ÖPIA) — Austria | http://www.oepia.at/ |
| Österreichischer Seniorenbund — Austria | http://www.seniorenbund.at/ |
| Pan Cyprian Volunteerism Coordinative Council — Cyprus | http://www.volunteerism-cc.org.cy/index.php?lang=en |
| PCOB — Netherlands | http://www.pcob.nl/ |
| Pensionistenverband Österreichs — Austria | http://pvoe.at/ |
| Pro Senectute — Switzerland | http://www.pro-senectute.ch/ |
| Provincial Caring Partnership | [www.violencepreventionb.org](http://www.violencepreventionb.org/) |
| Public Health Agency of Canada — Canada | http://www.phac-aspc.gc.ca/ |
| Public Legal Education and Information Service of New Brunswick | http://www.legal-info-legale.nb.ca/en/index.php?page=preventing_abuse_and_neglect_of_seniors#Safety_Planning |
| Public Safety Canada | http://www.publicsafety.gc.ca/ |
| Registered Nurses Association of Ontario | http://rnao.ca/ |
| Réseau Internet Francophone: Vieillir en liberté | http://www.rifvel.org/ |
| Resources Sharing Project | http://www.resourcesharingproject.org/ |
| Revera Inc. — Canada | http://reveraliving.com/ |
| Royal Canadian Mounted Police (RCMP) | [www.rcmp.ca](http://www.rcmp.ca/) |
| Scottish Intercollegiate Guidelines Network | <http://www.sign.ac.uk/> |
| Seniors Canada - Aînés Canada | http://www.seniors.gc.ca/eng/index.shtml |
| Seniors Citizens Care Foundation — Nigeria | http://www.sccfoundationng.org/ |
| Seniors Online Victoria | http://www.seniorsonline.vic.gov.au/ |
| Shehjaar Homes for Senior Citizens — India | http://www.shehjaarhomes.com/ |
| Slovenian Federation of Pensioners' Organisations (ZDUS) — Slovenia | http://www.zdus-zveza.si/ |
| Slovenska Filantropija (Slovene Philanthropy) — Slovenia | http://www.filantropija.org/en/ |
| Sourcewise Community Resource Solutions | http://www.mysourcewise.com/ |
| South African Older Persons' Forum — South Africa | http://www.saopf.org.za/ |
| South African Older Persons’ Forum | http://www.saopf.org.za/ |
| South Australia - Aged Rights Advocacy Service | http://www.sa.agedrights.asn.au/ |
| Spanish Red Cross | http://www.cruzroja.es/ |
| State Trustees Limited | http://www.statetrustees.com.au/ |
| Swiss Council of the Elderly (SSR) — Switzerland | http://www.ssr-csa.ch/ |
| Taiwan Association of Gerontology and Geriatrics | http://www.tagg.org.tw/ |
| Tasmania - Tasmanian Elder Abuse Helpline | http://www.advocacytasmania.org.au/elderabusehelpline.htm |
| Terra Nova — United States | http://www.terranova.org/ |
| The Alberta Elder Abuse Awareness Network (AEAAN) | http://www.albertaelderabuse.ca/index.cfm |
| The Andrus Gerontology Center | http://gero.usc.edu/ |
| The Association for the Aged (TAFTA) — South Africa | http://www.tafta.org.za/ |
| The Association of Care Giving Relatives and Friends/ Omaishoitajat ja Läheiser - Liitto ry — Finland | http://www.omaishoitajat.fi/ |
| The Catholic University of America — United States | http://www.cua.edu/ |
| The Drummond Foundation — Canada | http://www.drummondfoundation.ca/ |
| The European Federation of Older People (EURAG) | http://eurageurope.org/ |
| The Geneva Association — Switzerland | www.genevaassociation.org/ |
| The Hua Kwan Moral Society — Singapore | http://www.thkms.org.sg/services/ |
| The Monaco Gerontology Coordination Centre — Monaco | http://en.service-public-particuliers.gouv.mc/Social-health-and-families/Establishments/Seniors/The-Monaco-Gerontology-Coordination-Centre |
| The National Center for Victims of Crime | http://www.victimsofcrime.org/ |
| The National Longterm Care Ombudsman Resource Center | http://www.ltcombudsman.org/ |
| The National Social and Applied Gerontology Association | http://www.geroder.org/ |
| The Swedish Association for Seniors Citizens (SPF) — Sweden | http://spfpension.se/ |
| The Union for Senior Services (VALLI) — Finland | http://www.valli.fi/ |
| Toronto Police Service - Elder Abuse | http://www.torontopolice.on.ca/community/elderabuse.php |
| TSAO Foundation — Singapore | http://www.tsaofoundation.org/ |
| Turyak — Turkey | http://www.turyak.org/ |
| UN Committee for Human Rights — United Nations | http://www.ohchr.org/ |
| Unie KBO — Netherlands | http://www.uniekbo.nl/ |
| Unión Democrática de Jubilados y Pensionistas de España (UDP) — Spain | http://www.mayoresudp.org/portal/portada_dir/portada.aspx |
| Unión Estatal de Jubilados y Pensionistas de U.G.T. — Spain | http://www.jubiladosugt.org/ |
| Union Française des Retraités — France | http://www.retraites-ufr.com/ |
| United Nations Educational, Scientific and Cultural Organization (UNESCO) — United Nations | http://en.unesco.org/ |
| United Nations Second World Assembly on Ageing | http://www.un.org/en/events/pastevents/ageing_assembly2.shtml |
| US Department of Justice: Office of Justice Programs | http://www.ojp.gov/index.htm |
| US Department of Justice: Office on Violence Against Women | http://www.ovw.usdoj.gov/ |
| US National Guidelines Clearing House | <http://www.guideline.gov/> |
| Victoria - Seniors Rights Victoria | http://www.seniorsrights.org.au/ |
| Victorian Civil and Administrative Appeals Tribunal (VCAT) | http://www.vcat.vic.gov.au/ |
| Volunteers of America — United States | http://www.voa.org/Landing.aspx |
| Waterloo Region Committee on Elder Abuse | http://elderabusewaterloo.ca/ |
| Welsh Government | http://wales.gov.uk/?lang=en |
| Western Australia - Advocare | http://www.advocare.org.au/ |
| Wise and Healthy Aging (California Elder Justice Workgroup) | http://www.wiseandhealthyaging.org/ |
| Women Against Violence Europe (WAVE) | http://www.wave-network.org/ |
| Womenshealth.gov | <http://womenshealth.gov/aging/safety-abuse/elder-abuse.html> |
| World Demographic Association | http://www.wdaforum.org/ |
| World Health Organization (WHO) | http://www.who.int/en/ |
| Yad Sarah — Israel | http://www.yadsarah.org/ |
| Yang Memorial Methodist Social Service — China | http://www.yang.org.hk/e/default_home.asp |
| Život 90 — Czech Republic | http://eng.zivot90.cz/ |
